# Antifungal Activity and Potential Action Mechanism of Allicin against Trichosporon asahii

**DOI:** 10.1128/spectrum.00907-23

**Published:** 2023-05-18

**Authors:** Xin Yang, Shuang Bai, Jiamin Wu, Yunlong Fan, Yuekun Zou, Zhikuan Xia, Junhong Ao, Tong Chen, Mingwang Zhang, Rongya Yang

**Affiliations:** a Department of Dermatology, Yanbian University Hospital, Yanji, China; b Department of Dermatology, The Seventh Medical Center of PLA General Hospital, Beijing, China; c Department of Dermatology, Southwest Hospital, Army Medical University, Chongqing, China; University of Debrecen

**Keywords:** allicin, *Trichosporon asahii*, invasive trichosporonosis, COVID-19, biofilm, RNA-Seq, transcriptomics, oxidative stress

## Abstract

Trichosporon asahii is an emerging opportunistic pathogen that causes potentially fatal disseminated trichosporonosis. The global prevalence of coronavirus disease 2019 (COVID-19) poses an increasing fungal infection burden caused by T. asahii. Allicin is the main biologically active component with broad-spectrum antimicrobial activity in garlic. In this study, we performed an in-depth analysis of the antifungal characteristics of allicin against T. asahii based on physiological, cytological, and transcriptomic assessments. *In vitro*, allicin inhibited the growth of T. asahii planktonic cells and biofilm cells significantly. *In vivo*, allicin improved the mean survival time of mice with systemic trichosporonosis and reduced tissue fungal burden. Electron microscopy observations clearly demonstrated damage to T. asahii cell morphology and ultrastructure caused by allicin. Furthermore, allicin increased intracellular reactive oxygen species (ROS) accumulation, leading to oxidative stress damage in T. asahii cells. Transcriptome analysis showed that allicin treatment disturbed the biosynthesis of cell membrane and cell wall, glucose catabolism, and oxidative stress. The overexpression of multiple antioxidant enzymes and transporters may also place an additional burden on cells, causing them to collapse. Our findings shed new light on the potential of allicin as an alternative treatment strategy for trichosporonosis.

**IMPORTANCE** Systemic infection caused by T. asahii has recently been recognized as an important cause of mortality in hospitalized COVID-19 patients. Invasive trichosporonosis remains a significant challenge for clinicians, due to the limited therapeutic options. The present work suggests that allicin holds great potential as a therapeutic candidate for T. asahii infection. Allicin demonstrated potent *in vitro* antifungal activity and potential *in vivo* protective effects. In addition, transcriptome sequencing provided valuable insights into the antifungal effects of allicin.

## INTRODUCTION

Invasive fungal infections represent a global problem resulting in millions of deaths every year. In recent years, with the evolving coronavirus disease 2019 (COVID-19) pandemic around the world, the incidence of invasive fungal infections has been drastically rising. Common invasive fungal infections of COVID-19 patients are caused by Aspergillus spp., *Candida* spp., and *Mucormycetes* (1). It is noteworthy that recent investigations discovered that hospitalized COVID-19 patients had a high risk of developing invasive trichosporonosis, a rare yeast infection mainly caused by Trichosporon asahii (2, 3). Systemic infection with this emergent yeast is most often fatal. According to statistics reported by Benelli et al., COVID-19 patients with invasive trichosporonosis had an 86% mortality rate (4).

*Trichosporon* spp. are basidiomycetous yeast-like fungal pathogens that are widely distributed in nature and found in parts of the human body, such as the skin, gastrointestinal tract, and respiratory tract. T. asahii is the predominant pathogen of *Trichosporon* spp., which is able to cause life-threatening invasive trichosporonosis, particularly in immunosuppressed patients (5). The management of patients with trichosporonosis remains a challenge, as few antifungal drugs are effective against the genus *Trichosporon*. *In vitro* studies suggest that T. asahii exhibits intrinsic resistance to echinocandins and poor susceptibility to amphotericin B. In contrast, the triazole antifungals, such as fluconazole and voriconazole, have better *in vitro* and *in vivo* antifungal activities against T. asahii and have been selected as preferred drugs for systematic trichosporonosis (6, 7). Unfortunately, the widespread use of the azoles has contributed to the rapid emergence of azoles-resistant T. asahii isolates and even multidrug-resistant isolates, posing serious challenges to the clinical treatment of trichosporonosis (8, 9). Thus, the development of innovative therapeutic strategies is imperative. At present, natural compounds or natural bioactive products have attracted much attention in treating infectious diseases due to their broad-spectrum antimicrobial activity and infrequent inducible drug resistance (10, 11).

Allicin (diallyl thiosulfinate) is an important organosulfur compound found in garlic and other *Allium* species, with a broad range of biological activities, such as antibacterial, antifungal, antiparasitic, and antiviral activities (12). Regarding the effects of allicin on fungi, numerous studies have demonstrated its remarkable antifungal activity against various clinically important fungal species, including *Candida* spp., Cryptococcus spp., Aspergillus spp., *Trichophyton* spp., and others (13). However, the antifungal activity and action mechanism of allicin against *Trichosporon* spp. have not been defined.

The current study sought to assess the antifungal property of allicin against T. asahii and investigate its underlying action mechanisms.

## RESULTS

### Antifungal susceptibility.

Table 1 shows the *in vitro* susceptibility of T. asahii planktonic cells to allicin and antifungals. According to the tentative epidemiologic cutoff values (ECV_S_) for fluconazole (FCZ) and amphotericin B (AmB) (14), all strains (11/11) were susceptible to FCZ and the majority of strains (9/11) were resistant to AmB. The MICs of allicin against T. asahii varied significantly between 24 h and 48 h of incubation. The MIC_50_ and MIC_90_ ranges were 8 to 16 μg mL^−1^ and 16 to 64 μg mL^−1^ at 24 h and were 32 to 128 μg mL^−1^ and 64 to 256 μg mL^−1^ at 48 h. Allicin was highly active against all T. asahii strains, with the three clinical isolates exhibiting the greatest sensitivity (MIC_90_ of 64 μg mL^−1^) at the 24-h reading. The minimum fungicidal concentrations (MFCs) of allicin against T. asahii ranged from 128 μg mL^−1^ to 256 μg mL^−1^, with an average of 197.81 μg mL^−1^.

**TABLE 1:** MICs and MFCs of allicin and antifungals against *Trichosporon asahii*

Strains	Allicin concn (μg ml^−1^) at:					Fluconazole MIC_50_ (μg ml^−1^)	Amphotericin B MIC_100_ (μg ml^−1^)
	24 h		48 h				
	MIC_50_	MIC_90_	MIC_50_	MIC_90_	MFC		
CBS 2479	8	32	64	128	256	2	2
BMT 06-3-01	8	32	32	64	128	2	2
BMT 06-3-02	16	32	32	64	128	2	2
BMT 06-3-03	8	16	32	64	128	1	1
BMT 06-3-04	8	16	128	256	256	2	2
BMT 06-3-05	8	32	64	128	128	1	4
BMT 06-3-06	8	32	64	128	128	2	4
BMT 06-3-07	16	64	128	256	256	0.5	0.5
BMT 06-3-08	8	64	64	128	256	2	2
BMT 06-3-09	8	16	64	128	256	4	4
BMT 06-3-10	16	32	64	128	256	1	2

### Spot assays.

**FIG 1:**
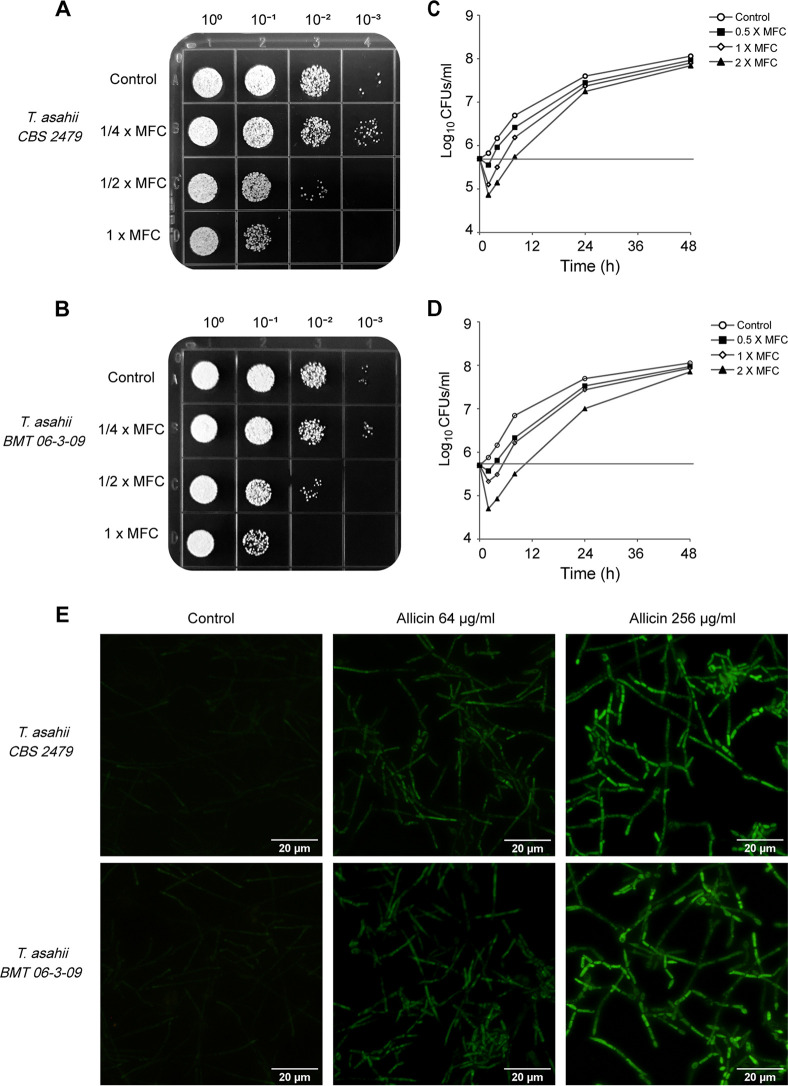
Spot assays (A and B) and time-kill curves (C and D) validated the inhibitory effect of allicin on T. asahii growth *in vitro*. (E) The intracellular ROS changes in T. asahii cells exposed to allicin were detected using the fluorescent probe DCFH-DA. Magnification, ×200.

Spot assays showed the efficacy of allicin against T. asahii strains in a visual form (Fig. 1A and B). Significant growth reduction was observed as the allicin concentration increased. The results showed an excellent correlation with the microdilution broth assay.

### Time-kill studies.

To further confirm the antifungal activity of allicin, a time-kill curve assay was carried out. As shown in Fig. 1C and D, allicin at 2× MFC showed distinct fungicidal activity during the first 8 h of incubation, with maximum reductions in viable counts reaching 1.02 log_10_ CFU mL^−1^ at 4 h for T. asahii CBS 2479 and 1.34 log_10_ CFU mL^−1^ at 8 h for T. asahii BMT 06-3-09. However, the full fungicidal end points (99.9% killing) could not be achieved with allicin throughout the incubation period of up to 48 h.

### Antibiofilm activity.

**FIG 2:**
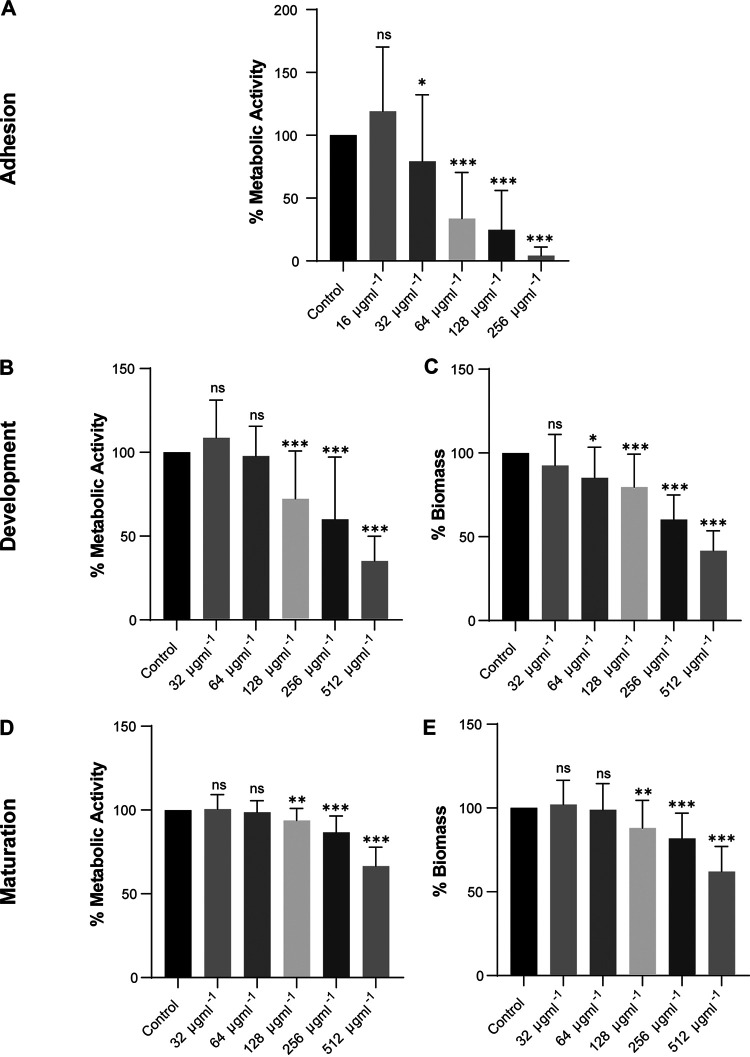
Effect of allicin on the metabolic activity and biomass of T. asahii (*n* = 6) biofilms in different growth stages, expressed as relative percentages of the absorbance of the XTT reduction assay (A, B, and D) and crystal violet staining (C and E), respectively. Adhesion of T. asahii cells after incubation for 6 h (A), after growth for 24 h (B and C), and in 48-h mature biofilms (D and E). ns, not significant; *, *P* < 0.05; **, *P* < 0.01; ***, *P* < 0.001 versus the control.

The results of the XTT [2,3-bis-(2-methoxy-4-nitro-5-sulfophenyl)-2H-tetrazolium-5-carboxanilide salt] reduction assay are shown in Fig. 2. Allicin inhibited the metabolic activity of T. asahii biofilms in a dose-dependent manner. Compared to the control, 32 μg mL^−1^ allicin inhibited biofilm adhesion by 20.8%, whereas 256 μg mL^−1^ allicin inhibited biofilm adhesion almost completely (Fig. 2A). However, with the development and maturity of biofilms, increased resistance to allicin was observed in T. asahii biofilm cells. Compared to the control, 256 μg mL^−1^ allicin inhibited biofilm activity by approximately 40.0% in the development stage and 13.3% in the maturation stage (Fig. 2B and D). The results of the crystal violet (CV) assay showed that in the presence of allicin, the biomass production of T. asahii biofilms was significantly reduced (Fig. 2C). Likewise, even at high concentrations, allicin exhibited limited activity against mature T. asahii biofilms. Allicin at 256 μg mL^−1^ caused a reduction in biomass of just 18.9% in mature biofilms compared with the untreated control (Fig. 2E).

**FIG 3:**
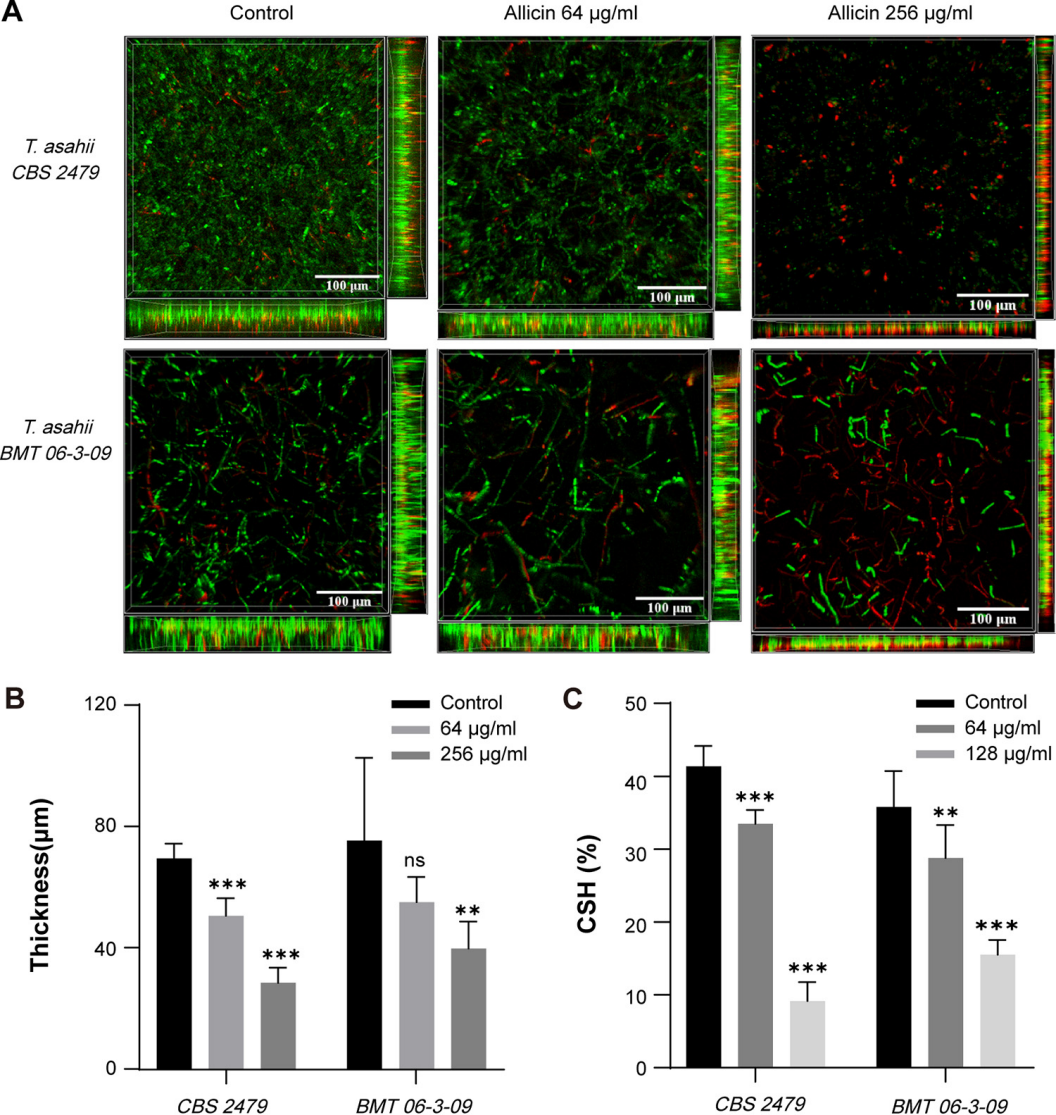
(A) CLSM images of T. asahii biofilms following treatment with allicin. SYTO 9 shows live cells (green), and PI shows dead or damaged cells (red). Magnification, ×400. (B) Quantitative analysis of biofilm thickness in CLSM images. (C) Effect of allicin on cell surface hydrophobicity of T. asahii (*n* = 3).

Figure 3A shows confocal laser scanning microscopy (CLSM) images of T. asahii biofilms, where live cells were stained green by SYTO 9 and dead/damaged cells were stained red by propidium iodide (PI). Control samples presented compact and dense biofilms. In contrast, allicin treatment resulted in a scattered biofilm structure dominated by dead cells (red) and significantly reduced biofilm thickness (Fig. 3B). The decrease in biomass was concomitant with a decline in biofilm cell viability, which corroborates the results of the XTT and CV assays.

### CSH.

The changes in cell surface hydrophobicity (CSH) of planktonic T. asahii cells were evaluated following exposure to allicin using a water-hydrocarbon two-phase assay. The results showed that allicin significantly decreased the cell surface hydrophobicity of T. asahii, with an 83.76% reduction observed at 128 μg mL^−1^ compared to the control group (Fig. 3C).

### ROS accumulation.

Intracellular levels of reactive oxygen species (ROS) in T. asahii were assessed by the dichlorodihydrofluorescein diacetate (DCFH-DA) assay (Fig. 1E). The T. asahii cells treated with allicin exhibited strong green fluorescence, whereas only weak fluorescence was observed in untreated cells. This suggests that allicin is able to increase accumulation of intracellular ROS.

### Ergosterol assay.

The effect of allicin on the T. asahii cell membrane was evaluated using the ergosterol assay. AmB was set as the positive control for ergosterol assay due to its antifungal action depending on interaction with fungal cell membrane ergosterol. The activities of allicin and AmB against T. asahii were significantly suppressed in the presence of ergosterol. For a large proportion of T. asahii strains (9/11), the MICs of AmB increased 2-fold or 4-fold in the presence of ergosterol. Similarly, an obvious increase in MICs of allicin was also observed for all T. asahii strains (11/11) in the presence of ergosterol (Table 2). The findings indicate that the antifungal activity of allicin is related to its effect on cell membrane ergosterol of T. asahii.

**TABLE 2:** MICs of allicin against *Trichosporon asahii* in the presence and absence of ergosterol at the 48-h reading^*a*^

Strain	MIC_50_ (μg ml^−1^) of allicin		MIC_90_ (μg ml^−1^) of allicin		MIC_100_ (μg ml^−1^) of AmB	
	Without ergosterol	With ergosterol	Without ergosterol	With ergosterol	Without ergosterol	With ergosterol
CBS 2479	64	>256	128	>256	2	8
BMT 06-3-01	32	128	64	>256	2	4
BMT 06-3-02	32	64	64	128	2	2
BMT 06-3-03	32	64	64	256	1	1
BMT 06-3-04	128	>256	256	>256	2	4
BMT 06-3-05	64	256	128	>256	4	4
BMT 06-3-06	64	>256	128	>256	4	8
BMT 06-3-07	128	>256	256	>256	0.5	2
BMT 06-3-08	64	256	128	>256	2	4
BMT 06-3-09	64	>256	128	>256	4	8
BMT 06-3-10	64	>256	128	>256	2	4

a Ergosterol was used at 200 μg ml^−1^.

### Sorbitol assay.

In addition, the effect of allicin on the cell wall of T. asahii was evaluated with a sorbitol assay. When fungi are exposed to drugs that act on the cell wall, sorbitol is capable of maintaining proper osmotic pressure. AmB was set as the negative control in sorbitol assay. The antifungal activity of AmB is unlikely affected by sorbitol, in view of its mode of action (15). Unexpectedly, the MICs of AmB increased significantly in the presence of sorbitol compared to the control (data not shown). A likely explanation is that the sorbitol reduces total ergosterol of T. asahii cells (16). As a result, evidence of the effect of allicin on the T. asahii cell wall was not able to be obtained by the sorbitol assay.

### Morphology and ultrastructure.

**FIG 4:**
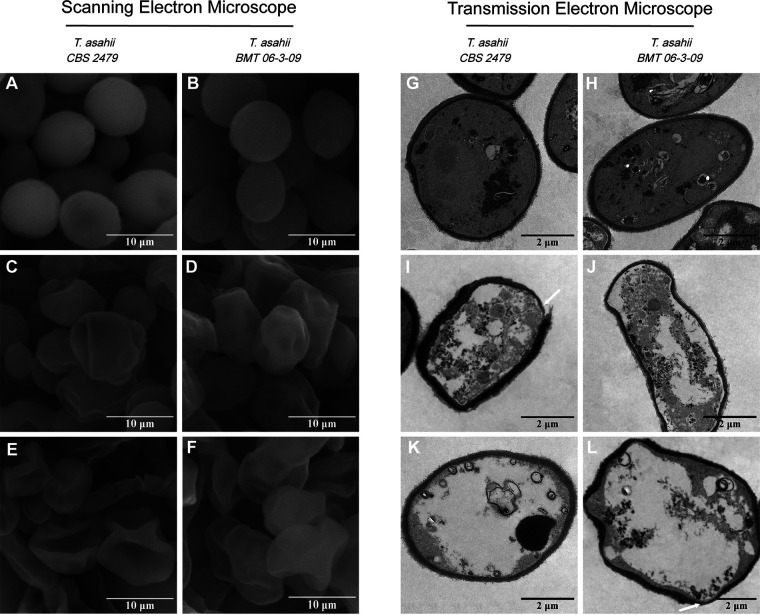
SEM and TEM images of T. asahii cells. (A, B, G, and H) Control cells. (C to F) The allicin-treated cells became irregular, pitted, and shriveled in SEM images. Notable structural disorganization within the cytoplasm (I and J), obvious vacuolation (K and L), nonuniform thickness, irregular plasma membrane, and degraded cell wall (white arrows) were observed in allicin-treated cells by TEM. Magnifications, ×7,000 for SEM and ×3,000 for TEM.

The effect of allicin on the morphology of T. asahii was observed using scanning electron microscopy (SEM) (Fig. 4A to F). The untreated cells possessed normal shapes and smooth surfaces, while allicin-treated T. asahii cells exhibited rough pitted surfaces and a severely shriveled appearance.

The ultrastructure changes of T. asahii cells were examined using transmission electron microscopy (TEM) (Fig. 4G to L). Untreated cells exhibited complete cell walls, plasma membranes, and organelles. In contrast, allicin caused significant cellular damage, including structural disturbances with nonuniform thickness, irregular plasma membranes and obvious vacuolation.

### Anti-T. asahii activity *in vivo*.

**FIG 5:**
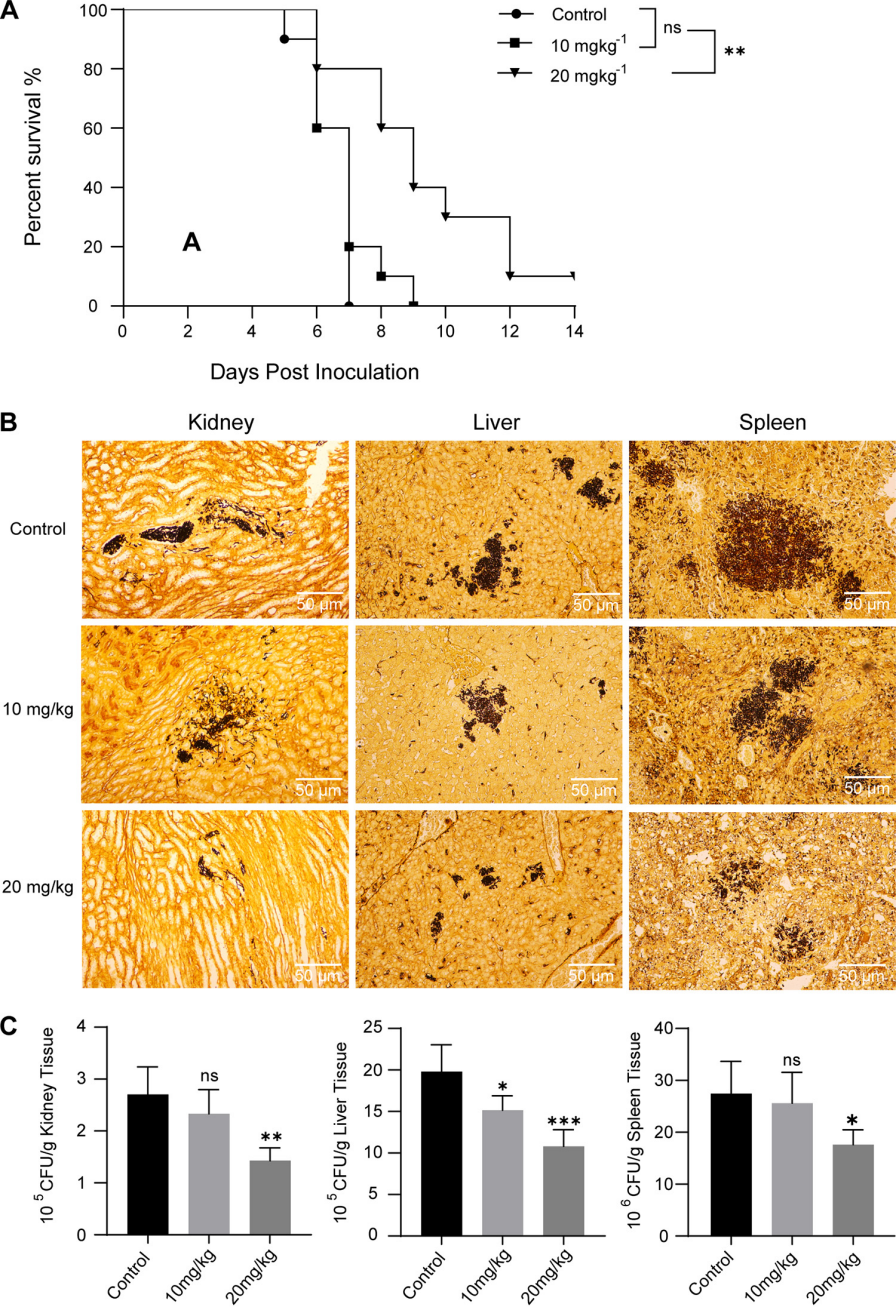
(A) Survival curves in mice inoculated intravenously with T. asahii BMT 06-3-09 and treated with physiological saline (i.p.) or allicin (i.p.). Treatment started 3 days before T. asahii-induced infections and lasted until 4 days postinfection. Mice were monitored until day 14 postinfection. Each group consisted of 10 mice for survival analysis. (B) Tissue fungal burden of mice was visualized using GMS stain at 5 days postinfection. Fungal cells were stained black by GMS. Magnification: ×200. (C) Quantitative analysis of tissue fungal burden, expressed as CFU per gram. ns, not significant; *, *P* < 0.05; **, *P* < 0.01; ***, *P* < 0.001 versus control.

The *in vivo* protective effect of allicin was evaluated in the murine model with systemic trichosporonosis. Experimental mice were treated with allicin or saline for 7 consecutive days. The final results showed that the mean survival time (MST) was 6.5 days in saline-treated mice; in contrast, treatment with 20 mg kg^−1^ allicin prolonged MST of mice to 9.4 days (*P* < 0.01). However, there were no significant differences in MST of mice between the group receiving 10 mg kg^−1^ allicin (MST of 6.9 days) and the saline group (*P > *0.05). By the end of the trial, one mouse was still alive in the group receiving 20 mg kg^−1^ allicin, whereas all mice in the other two groups died (Fig. 5A).

The severity of T. asahii infection was assessed by determining the tissue fungal burden of mice from various experimental groups. Quantification of fungal burden in the control group revealed that the spleen (7.43 ± 0.11 log_10_ CFU/g) was the most severely involved tissue, followed closely by liver (6.29 ± 0.07 log_10_ CFU/g) and kidney (5.43 ± 0.09 log_10_ CFU/g). Compared to the control, a significant reduction in tissue fungal burden was observed in the group receiving 20 mg kg^−1^ allicin (47.19% reduction in kidney [*P* < 0.01]; 45.42% reduction in liver [*P* < 0.001]; 35.83% reduction in spleen [*P* < 0.05]) (Fig. 5B and C).

### Transcriptome sequencing (RNA-Seq) quality and mapping rate.

A total of 245,150,678 raw reads with adapters and low-quality reads were identified. Following quality control, 1,403,742 reads were filtered out, leaving 243,746,936 high-quality clean reads for further analysis. The rRNA mapped reads were removed before read alignment, and approximately 89.22% of the paired-end clean reads were mapped to the reference genome, containing approximately 88.34% uniquely mapped reads. Table 3 summarizes the information presented above.

**TABLE 3:** RNA-Seq quality and mapping rate

Sample	No. of reads		No. (%) mapped	
	Raw	Clean	Unique	Total
Treatment-1	37,422,164	37,208,550	31,966,951 (88.31)	32,318,614 (89.28)
Treatment-2	40,934,342	40,685,208	35,090,109 (88.02)	35,454,883 (88.93)
Treatment-3	40,951,936	40,725,356	34,569,483 (88.41)	34,935,426 (89.35)
Control-1	41,914,734	41,659,424	36,443,554 (88.06)	36,753,254 (88.81)
Control-2	44,627,768	44,364,542	38,772,765 (88.24)	39,138,560 (89.07)
Control-3	39,299,734	39,103,856	34,611,277 (89.03)	34,930,701 (89.86)

### DEG analysis.

**FIG 6:**
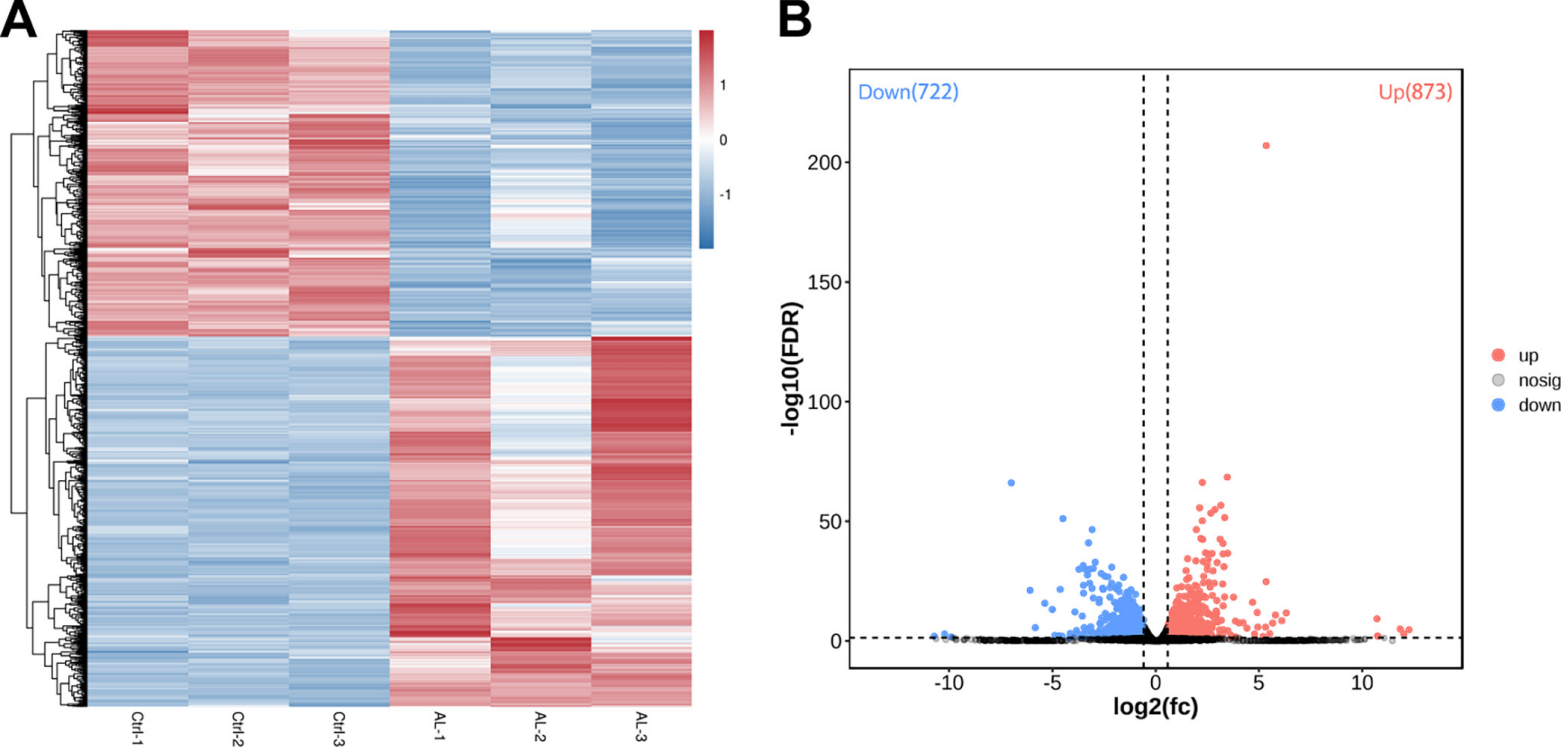
Transcriptome differences induced by allicin in T. asahii CBS 2479. (A) Heat map of DEGs. Hierarchical clustering analysis of DEGs according to the color scale indicates gene expression changes. (B) Volcano plot of DEGs. Red represents upregulated DEGs, and blue represents downregulated DEGs.

In this study, the transcriptome expression profiles of T. asahii were significantly affected under allicin stress. A total of 1,595 genes were identified as differentially expressed genes (DEGs) according with the filter criteria (absolute value of fold change [|FC|] ≥ 1.5; false discovery rate [FDR] < 0.05). Among these DEGs, 873 were upregulated and 722 were downregulated in the allicin-exposed samples compared to the untreated samples. The difference in quantity between upregulated and downregulated genes was less than 10% of total DEGs. Hierarchical cluster analysis of DEGs revealed intragroup coherence and intergroup difference, indicating that the data were highly reliable (Fig. 6A and B; also, see Table S1 in the supplemental material).

### Functional enrichment analysis.

To obtain further insight into biological responses of T. asahii to allicin, a functional enrichment analysis, including GO (Gene Ontology) functional categories and KEGG (Kyoto Encyclopedia Genes and Genomes) pathways, was performed on the up- and downregulated genes.

**FIG 7:**
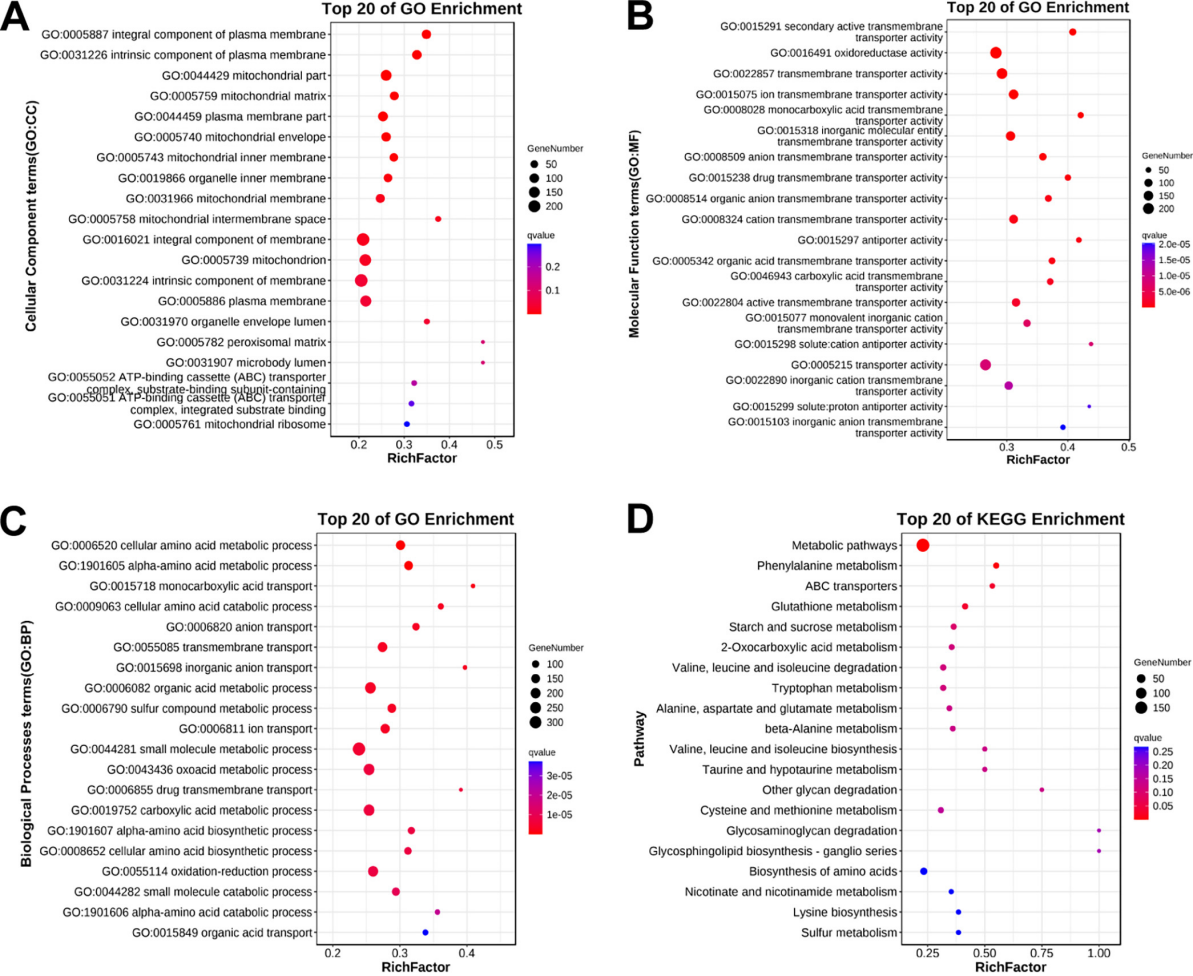
GO enrichment analysis and KEGG pathway analysis. (A) Top 20 cellular component terms in GO categories. (B) Top 20 molecular function terms in GO categories. (C) Top 20 biological processes terms in GO categories. (D) Top 20 KEGG pathways.

A total of 1,045 DEGs (65.5% of total) were mapped to three GO categories with 116 GO terms, including 15 cellular component terms (GO: CC), 54 molecular function terms (GO: MF), and 47 biological processes terms (GO: BP). In the cellular component category, “plasma membrane” and “mitochondrion” were the principal enriched components. In the molecular function category, “catalytic activity (GO:0003824),” “oxidoreductase activity (GO:0016491),” “cofactor binding (GO:0048037),” and “transporter activity (GO:0005215)” were important enriched terms. Terms of small-molecule metabolic and biosynthetic process were significantly enriched in the biological processes category, including “organic acid metabolic process (GO:0006082),” “drug metabolic process (GO:0017144),” “cellular amino acid biosynthetic process (GO:0008652),” and so on (Fig. 7A to C; Table S2).

In KEGG enrichment analysis, 303 DEGs (19.0% of total) were mapped to 5 KEGG categories with 107 pathways, including 1 organismal system term, 3 environmental information processing terms, 8 cellular process terms, 19 genetic information processing terms, and 76 metabolism terms, without considering significance levels. Some pathways associated with signal transduction (such as MAPK signaling pathway [ko04011] and phosphatidylinositol signaling system [ko04070]), energy metabolism (such as oxidative phosphorylation [ko00190]), amino acid metabolism (such as tryptophan metabolism [ko00380], cysteine and methionine metabolism [ko00270], and glycine, serine, and threonine metabolism [ko00260]), lipid metabolism (such as steroid biosynthesis [ko00100] and biosynthesis of unsaturated fatty acids [ko01040]), membrane transport (such as ABC transporters [ko02010]), and gene replication, transcription, and translation (such as DNA replication [ko03030], nucleotide excision repair [ko03420], RNA polymerase [ko03020], and ribosome [ko03010]) were impacted by treatment with allicin (Fig. 7D; Table S3).

In general, the functional enrichment analysis showed that large numbers of complex cellular elements and pathways are involved in the response of T. asahii to allicin stress.

### PHI-base analysis.

PHI-base (Pathogen-Host Interactions Database) is a multispecies phenotype database devoted to exploration of pathogen-host interactions. In this study, the 335 DEGs (21% of total) were mapped to 6 phenotype categories: reduced virulence (55.22%, 185/335), unaffected pathogenicity (27.46%, 92/335), loss of pathogenicity (12.84%, 43/335), lethality (2.99%, 10/335), increased virulence (hypervirulence) (1.19%, 4/335), and chemistry target: resistance to chemical (0.30%, 1/335) (Table S6).

### Validation of RNA-Seq through qRT-PCR analysis.

**FIG 8:**
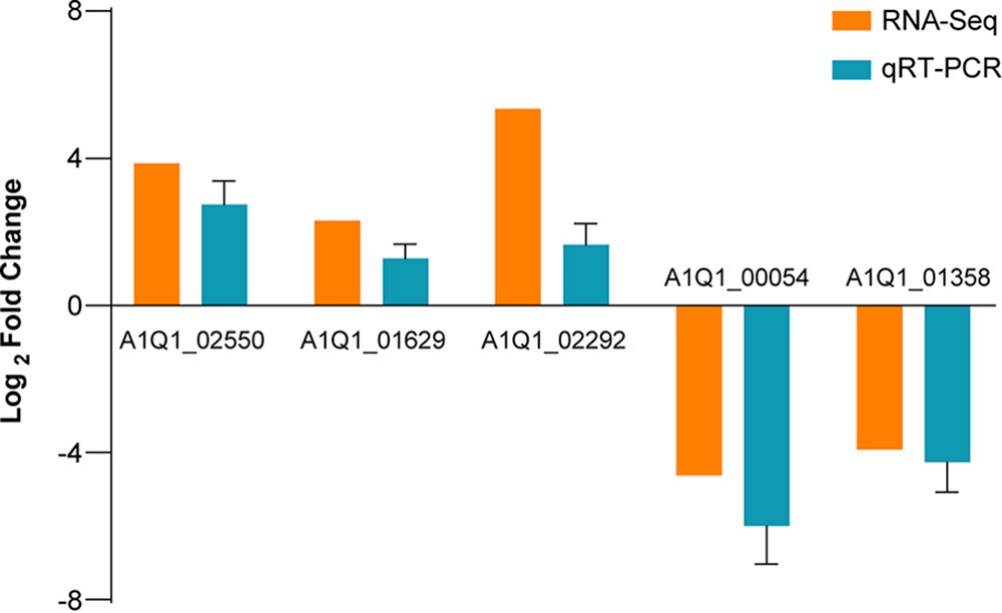
qRT-PCR analysis to validate the genes in RNA-Seq data. Relative expression level of DEGs is shown using an index of log_2_ fold change.

To validate the expression levels of the DEGs identified in the transcriptomic analysis, five genes were selected to confirm their expression using quantitative reverse transcription-PCR (qRT-PCR). As shown in Fig. 8, the congruent expression trends observed between qRT-PCR and RNA-Seq analysis serve to validate the reliability of our transcriptome data.

## DISCUSSION

Currently, only a few antifungal agents are approved for the treatment of invasive fungal infections: azoles, polyenes, and echinocandins (17). The emergence of resistant strains complicates patient management. Thus, there is a pressing need for novel antifungal agents. Medicinal plants have been proven to be an invaluable resource for exploring pharmacologically active molecules. In recent years, a large number of medicinal plants or their derivatives have undergone examination for their pharmacological activities. Garlic (Allium sativum L.) is an aromatic herbaceous plant whose extracts and isolated compounds possess various biological activities, including anticarcinogenic, antiparasitic, antibacterial, and antifungal activities. Allicin is the most biologically active sulfur-containing compound in garlic (18, 19). The purpose of this study was to investigate the activities and mechanisms of allicin against T. asahii.

In this study, *in vitro* susceptibility testing demonstrated that allicin was highly active against T. asahii. Although allicin has been reported to exhibit significant inhibitory activity against various microorganisms, MICs of allicin vary slightly among different species (20–22). In addition to species differences, the discrepancies in MICs for allicin may be also attributed to differences in drug sources and antifungal susceptibility testing assays. Moreover, the effectiveness of allicin against T. asahii was further confirmed by a spot assay and a time-kill study. It is noteworthy that all tested concentrations of allicin were unable to reach the full fungicidal endpoints (99.9% killing). This demonstrates that allicin lacks potent fungicidal effect on T. asahii. Another observation was the inhibitory effect of allicin on T. asahii biofilms. The formation of biofilms plays an important role in the pathogenesis of *Trichosporon* catheter-related bloodstream infections and poses significant challenges for treatment and eradication, often leading to persistent or recurrent infections despite treatment with antifungals (7, 23). In this research, allicin significantly inhibited the growth of nascent T. asahii biofilms while only weakly inhibiting mature biofilms. The antibiofilm activity of allicin has been demonstrated in multiple microorganisms, such as Candida albicans, Staphylococcus aureus, Escherichia coli, and Proteus mirabilis (24–26). Researchers also found that allicin could exert antibiofilm effects by downregulating the expression of key genes related to biofilm formation. For example, Khodavandi et al. (27) reported that allicin significantly decreased the gene expression of HWP1, which has been proved to be essential for Candida albicans biofilm adherence (28). However, in the current study, we were unable to verify the expression of biofilm-related genes as other researchers have done, as no studies have been performed to identify specific genes or biological pathways related to T. asahii biofilms. Therefore, the intricate molecular mechanism underlying allicin-mediated antibiofilm activity remains to be further studied.

Surface adhesion is regarded as an important first step in the biofilm formation. The intrinsic high-level cell surface hydrophobicity of T. asahii cells is responsible for their rapid surface adhesion (29, 30). To explore the effect of allicin on T. asahii surface adhesion, a CSH assay was performed in this study. The results showed that allicin treatment remarkably reduced surface hydrophobicity of T. asahii cells, thus interfering with the cell-substratum interaction, which provided a possible explanation for the inhibitory effects of allicin on T. asahii biofilm adhesion.

*In vivo* antifungal activity of allicin has also been demonstrated in previous studies. In this study, allicin was administered intravenously to mice 3 days before T. asahii-induced infections and treatment lasted until 4 days postinfection. The survival analysis results showed that the MST of saline-treated mice was 6.5 days postinfection, whereas the MST of mice receiving 20 mg kg^−1^ allicin was 9.4 days (*P* < 0.01). Meanwhile, 20 mg kg^−1^ allicin significantly reduced the fungal burden in kidney (*P* < 0.01), spleen (*P* < 0.05), and liver (*P* < 0.001), compared to the control. These findings suggest a superior protective effect of allicin *in vivo*. Furthermore, our previous investigation demonstrated the absence of mortality and significant visceral damage in uninfected mice subjected to 20 mg kg^−1^ allicin treatment for 1 week, thus highlighting the safety profile of allicin at this dosage (Fig. S1). Koch et al. reported that intravenous injection of allicin at 60 mg kg^−1^ is lethal for mice (31). As we know, the survival rate of mice can be affected by several major factors, such as T. asahii virulence, efficacy of immunosuppression, and dosages of allicin. Based on the evidence presented above, further research is required to investigate whether higher dosages of allicin can further prolong survival of mice with systemic trichosporonosis, assuming that it is safe and nontoxic.

In our study, GO enrichment analysis showed that the plasma membrane was one of the most enriched components, implying that composition and integrity of the plasma membrane of T. asahii are greatly affected by allicin. Further analysis found that several genes encoding essential enzymes in the ergosterol biosynthesis pathway (Table S4), such as C-4 methyl sterol oxidase (ERG25), sterol 24-C-methyltransferase (ERG6), and lanosterol 14-alpha-demethylase (ERG11), were downregulated, indicating that allicin caused a reduction in ergosterol content. Ergosterol, an important component of fungal plasma membranes, plays an essential role in maintaining a variety of cellular functions, including membrane integrity, membrane fluidity, and membrane-associated proteins activity, and has always been an important target for antifungal-drug development. On the other hand, the ergosterol assay suggested that allicin may interact with plasma membrane ergosterol. In addition, oxidative stress induced by allicin was capable of disrupting plasma membrane via phospholipid peroxidation (20). Previous cytological researches confirmed the effects of allicin on fungal plasma membrane. Ogita et al. found that in Saccharomyces cerevisiae, allicin enhanced fungicidal activity of AmB by inhibiting ergosterol trafficking from the plasma membrane to the vacuole membrane (32). Li et al. recently reported that allicin exerted antifungal efficacy against Cryptococcus neoformans by regulating plasma membrane-related pathways to block the cell membrane (33). The effects of allicin on fungal cell membrane were also reported by Khodavandi et al., Aala et al., and Kim et al. (27, 34, 35).

The cell wall is indispensable for maintaining fungal cellular homeostasis, as it serves to safeguard the cell against osmotic stress and mechanical injury while also contributing to the establishment of cellular morphology. In fact, the cell wall has been an important antimicrobial target due to its absence in human cells. In a recent study, researchers determined the effect of allicin on Staphylococcus aureus cell wall by matrix-assisted laser desorption ionization–time-of-flight (MALDI-TOF) mass spectrometry and identified Fem enzymes as a potential target for allicin in peptidoglycan synthesis (36). In addition, a study on Aspergillus spp. indicated that allicin could selectively target cell wall biosynthesis, thereby disrupting the structural integrity of the cell wall (37, 38). In present study, allicin was found to inhibit the expression of genes encoding 1,3-beta-glucan synthase and chitin synthase (Table S4), which are mainly responsible for regulating biosynthesis of glucan and chitin, two essential components of the fungal cell wall. In addition, genes encoding glycosylphosphatidylinositol (GPI)-anchored protein were also observed to be downregulated (Table S4). The GPI-anchored proteins participate in a variety of cellular processes in fungal pathogens (39, 40). Most notably, they mediate posttranslational modification related to plasma membrane and cell wall organization, leading to the transportation of diverse proteins from the endoplasmic reticulum to the cell wall and ensuring the integrity of the cell wall. Antifungals targeting the GPI anchor synthesis pathway have been developed and have shown remarkable fungistatic activity in mice with disseminated fungal infection (41). We speculate that allicin-induced damage to plasma membranes and cell walls may increase cell permeability, leading to an osmotic imbalance and ultimately resulting in cell inflation and collapse. SEM and TEM observations further confirmed degradation and disturbance of T. asahii cell structures caused by allicin.

The mitochondrion is another principal enriched component after exposure to allicin. Mitochondria are the major energy production centers in eukaryotes. Further analysis revealed that the majority of genes related to the mitochondrial respiratory chain and oxidative phosphorylation were upregulated (Table S4). Among them, cytochrome *c* oxidase and NADH-ubiquinone oxidoreductase participate in the formation of the respiratory chain complex, which serves as a crucial site for ATP production by coupling oxidation with phosphorylation in mitochondria (42). This implies an immediate requirement for energy to repair cellular damage and sustain regular cellular activity in response to allicin exposure of T. asahii. However, the genes encoding pyruvate dehydrogenase (Table S4), such as pyruvate dehydrogenase (E1) and dihydrolipoyl transacetylase (E2), were downregulated following allicin treatment. The pyruvate dehydrogenase complex catalyzes oxidation decarboxylation of pyruvate to produce acetyl coenzyme A (acetyl-CoA), which is a key step in aerobic oxidation of glucose and serves as a critical link between glycolysis and the tricarboxylic acid cycle (43). Thus, it is postulated that inhibition of glucose catabolism may be one of the potential antifungal mechanisms of allicin, leading to energy deficiency and ultimately accelerating cell death. The mitochondrial respiratory chain is the main source of ROS. In the process of electron transfer, leaked electrons are picked up by oxygen to produce ROS (44). DCFH-DA analysis showed that allicin-treated T. asahii cells exhibited a high level of intracellular ROS accumulation. ROS play an important role in the regulation of cell survival. The moderate levels of ROS promote cell proliferation and survival. In contrast, excessive ROS induce oxidative stress, leading to oxygenation damage, such as DNA strand breakage, protein degeneration, phospholipid peroxidation, and even cell death (45, 46). Among these, plasma membrane phospholipids are regarded as a target of oxidative stress, which helps to partly explain the plasma membrane damage caused by allicin. To respond to oxidative stress induced by allicin, antioxidant systems were activated in T. asahii cells, resulting in upregulation of genes encoding catalase (CAT), glutathione peroxidase (GPx), glutathione reductase (GR), peroxidase (PO), and superoxide dismutase (SOD) (Table S4). These antioxidant enzymes together constitute the cellular defense system to mitigate intracellular ROS accumulation and maintain redox homeostasis (47).

Another important finding is that allicin increased the expression of ATP-binding cassette (ABC) transporter-related genes (Table S4), such as the YOR1, Pdr11, and MDR1 genes. The ABC transporters are present in cellular and intracellular membranes and are involved in the import and removal of substances from cells and tissues, including drug transmembrane transport (48). This suggests that drug efflux systems mediated by ABC transporters were activated in T. asahii cells to decrease intracellular allicin concentrations and protect cells from allicin stress.

KEGG analysis showed a general upregulation of genes involved in replication, repair, transcription, and translation pathways (Table S5). As a compensatory mechanism, protein synthesis is strengthened, resulting in increased protein production to maintain normal cell activities and repair cell damage caused by allicin stress. Nevertheless, with mitochondrial dysfunction, the enhancement of cell damage repair and drug efflux may place an additional burden on T. asahii cells, accelerating cells collapse.

Aside from GO and KEGG analysis, we discovered that a small portion of DEGs were annotated in PHI-base (Table S6), implying potential effects of allicin on the pathogenicity and virulence of T. asahii. However, these details remain unclear and require extensive investigation (49).

We aware that our research has some limitations. First, specific targets of allicin have yet to be verified, and this will be the focus of our follow-up research. Second, researchers proposed that allicin may function better in combination with antifungal drugs than when used alone (50). Thus, more studies need to be carried out to determine the *in vivo* and *in vitro* antifungal activities of allicin in combination with azoles against T. asahii in future work.

In conclusion, allicin treatment effectively inhibited T. asahii planktonic cells growth, interfered with biofilm formation, and exhibited positively protective effects in mice with systemic trichosporonosis, indicating the high potential of allicin as a potent antifungal candidate for treatment of T. asahii infection. Electron microscopy and biochemical analysis showed that cellular morphology and ultrastructure were damaged and ROS overproduction was induced following exposure to allicin, suppressing the growth of T. asahii. In addition, based on RNA-Seq analysis, the changes in transcriptome profiling of T. asahii following exposure to allicin were first revealed in our study, investigating underlying action mechanisms of allicin at the molecular level.

## MATERIALS AND METHODS

### Strains.

In this study, a total of 11 T. asahii strains were used, including the type strain CBS 2479 and 10 common clinical isolates. All isolates were identified based on sequencing of the IGS1 region (51). The strains were routinely stored at −80°C. Before the start of an experiment, strains were plated on potato dextrose agar (PDA) medium to sustain optimal vitality through passage cultivation at 35°C.

### Determination of MICs.

The microdilution broth method described in CLSI document M27-A3 was employed for *in vitro* antifungal susceptibility testing (52). The stock solutions of allicin (50 mg mL^−1^) and AmB (3.2 mg mL^−1^) were prepared in dimethyl sulfoxide (DMSO; Sigma, USA), while FCZ (1.28 mg mL^−1^) was dissolved in sterile distilled water. The stock solutions were then diluted with RPMI 1640 medium to prepare the working solution. The highest final concentrations used in 96-well polystyrene plates were 256 μg mL^−1^ for allicin (MCE, Shanghai, China), 128 μg mL^−1^ for FCZ (Sigma, USA), and 16 μg mL^−1^ for AmB (Sigma-Aldrich, USA). The MICs were determined as the lowest drug concentrations required to inhibit visible T. asahii growth. After 48 h of incubation, MIC_50_s of FCZ and MIC_100_s of AmB were determined. The MIC_50_s and MIC_90_s of allicin against T. asahii were determined after 24 and 48 h of incubation, respectively.

### Determination of MFCs.

The MFCs were determined by the method reported by Cantón et al. (53). Briefly, a 100-μL suspension from each MIC well was spread onto PDA plates and incubated at 35°C for 48 h. The MFC was defined as the minimum concentration that killed 99.9% of the inocula.

### Spot assay.

Overnight cultures of T. asahii in yeast-extract peptone dextrose (YPD) medium were collected, washed, and resuspended in saline solution (1 × 10^7^ CFU ml^−1^). Serial 10-fold dilutions supplemented with allicin were transferred to 96-well plates and incubated for 8 h at 30°C and 300 rpm. Then, 20-μL aliquots of each well were spotted on PDA plates and cultured overnight at 35°C. The T. asahii colonies were photographed at the end of incubation.

### Time-kill studies.

Experiments were conducted as suggested by Cordeiro et al. with adaptations (54). The T. asahii yeast suspension (5 × 10^5^ CFU ml^−1^) was incubated in RPMI 1640 medium (pH 7.0 with 0.165 M morpholinepropanesulfonic acid [MOPS]) supplemented with allicin (0.5×, 1×, and 2× MFC) at 35°C. At specified time points (0, 2, 4, 8, 24, and 48 h), 10-fold serial dilutions of suspension were streaked onto PDA plates, and the number of CFU was calculated after an additional 48 h of incubation at 35°C. The minimum detectable level was set at 100 CFU/mL. Growth controls were performed using RPMI medium without drugs. At least three independent experiments were conducted.

### Biofilm assay.

Overnight cultures of T. asahii grown in YPD medium were collected, washed, and resuspended in RPMI 1640 medium to 1 × 10^6^ CFU mL^−1^ for inoculation. Aliquots (200 μL per well) of inoculum were then dispensed into flat 96-well polystyrene plates and incubated at 35°C. T. asahii cells adsorbed to the bottom of plates after 6 h of incubation, marking the adhesion stage of biofilms. The cells were then washed gently with sterile phosphate-buffered saline (PBS) before an additional 24 h and 48 h of incubation, representing the development and maturation stages of biofilms, respectively. Allicin was administered to biofilms cells at various stages, with the highest final concentrations of 256 μg mL^−1^ for biofilms cells in the adhesion stage and 512 μg mL^−1^ for those in the development and maturation stages. Metabolic activity and total biomass of T. asahii biofilms were evaluated using the XTT reduction assay (55) and the crystal violet assay (56), respectively. Untreated T. asahii biofilm cells served as the control. Biofilm assays were conducted in triplicate.

### Cell surface hydrophobicity assay.

The CSH experiment was performed using a water-hydrocarbon two-phase assay, as described by Kurakado et al. with adaptations (29). Briefly, T. asahii strains were cultured in YPD medium at 35°C overnight. Overnight cultures were transplanted into RPMI 1640 medium supplemented with allicin and incubated for 8 h at 30°C with shaking at 120 rpm. Subsequently, T. asahii cells were collected, washed, and resuspended in sterile PBS to an *A*_600_ of 1.0. Then, 1.2-mL aliquots of suspension were mixed with 0.3 mL octane (Damao, Tianjin, China) and incubated for 15 min incubation at 37°C in a water bath. The mixture was then vortexed for 5 min and allowed to stand until two phases separated. The absorbances of the aqueous phase were measured at 630 nm, with aliquots without octane overlay serving as the control. The relative cell surface hydrophobicity was calculated as follows: [(*A*_630_ of the control − *A*_630_ of octane overlay)/*A*_630_ of the control] × 100. Experiments were performed in triplicate.

### ROS assay.

The ROS level was assessed as described by Lei et al.(57). T. asahii suspensions (5 × 10^5^ CFU mL^−1^) in RPMI 1640 medium were plated in 24-well plates and incubated for 6 h at 35°C, followed by exposure to allicin for 6 h at 30°C. T. asahii cells were then stained with 10 μmol L^−1^ DCFH-DA (Goyoo, China) for 20 min at 35°C. After that, T. asahii cells were washed with sterile PBS to remove remaining DCFH-DA and then observed under a fluorescence microscope at 488 nm. Untreated cells served as the control.

### Ergosterol assay and sorbitol assay.

The ergosterol and sorbitol assays were performed according to the procedure described by Turecka et al. with adaptations (58). For ergosterol assays, ergosterol (Psaitong, China) was first dissolved in trichloromethane and then diluted with RPMI 1640 medium. The MICs of allicin against T. asahii were determined using the broth microdilution method, as previously described, in the presence or absence of exogenous ergosterol. The ergosterol concentration in 96-well plates was finally adjusted to 200 μg mL^−1^, with AmB serving as the positive control. For sorbitol assays, sorbitol (Psaitong, China) was dissolved directly in RPMI 1640 medium and filtered through 0.22-μm filters after being adjusted to a concentration of 1.6 M. The MICs of allicin against T. asahii were determined in the presence or absence of sorbitol with a final concentration of 0.8 M. AmB was used as a negative control. In both studies, the MICs were evaluated at 48 h. Each treatment was carried out in triplicate.

### SEM.

The overnight cultures of T. asahii in YPD medium were collected and adjusted to 1 × 10^7^ CFU mL^−1^ in sterile saline solution. Subsequently, the T. asahii cell suspension was treated with allicin (0× or 1× MFC) and incubated for 12 h at 30°C. Following fixation and dehydration procedures outlined by Lan et al. (59), cell images were captured using a TM3000 SEM (Hitachi, Japan).

### TEM.

The allicin treatment used for SEM analysis was also employed for TEM analysis of T. asahii cells, with fixation and dehydration procedures described by Turecka et al. (58). The ultrastructure of T. asahii cells was observed using a CM100 TEM (Philips, Netherlands).

### CLSM.

CLSM analysis was used to assess the effects of allicin on the structure and viability of biofilms. T. asahii cells were cultured in RPMI 1640 medium at 35°C for 6 h in 24-well polystyrene plates, resulting in the adhesion stage of biofilms. The plates were then washed with sterile PBS before biofilm cells were treated by allicin for 24 h at 30°C. After that, the biofilms were stained with a LIVE/DEAD BacLight bacterial viability kit (Thermo Fisher Scientific, USA) for 20 min at 35°C. Viable cells stained with SYTO 9 were observed at 485 nm using a TCS-SP8 CLSM (Leica, Germany), while dead and damaged cells stained with propidium iodide (PI) were observed at 535 nm.

### Murine systemic trichosporonosis model.

Male 6- to 8-week-old ICR mice (20 to 22 g) were obtained from SPF Biotechnology (Beijing, China) and housed in standard cages with *ad libitum* access to water and food. Mice were kept at 21 to 23°C under an appropriate light period. Before the experiments, mice were fed for 1 week to allow them to adapt to the new environment. Animal experiments were approved by the animal ethics committee of the Seventh Medical Center of PLA General Hospital, with ethics number no. 145 in 2022.

Prior to T. asahii inoculation, mice were first immunosuppressed for 3 days with a single intraperitoneal (i.p.) injection of cyclophosphamide (Endoxan, Germany) at a dose of 200 mg kg^−1^ per day. The yeast suspension of T. asahii BMT 06-3-09 was then prepared and adjusted in physiological saline to 10^7^ CFU mL^−1^. In order to induce systemic infection, yeast suspension was injected into the lateral tail vein at a dose of 0.2 mL per mouse. Mice were randomly divided into three groups (*n* = 15). The experimental groups received allicin via lateral tail vein injection at doses of either 10 mg kg^−1^ or 20 mg kg^−1^, while the control groups received an equivalent volume of physiological saline (0.2 mL per mouse). For survival analysis, 10 mice were allocated to each group and monitored daily until day 14 postinoculation. Tissue fungal burden analysis was conducted using five mice per group, which were sacrificed at 5 days postinoculation. Spleens, kidneys, and livers were aseptically removed, weighed, and homogenized in 1 mL sterile saline. Serial 10-fold dilutions of the homogenates were plated on PDA and incubated for 48 h at 35°C. The numbers of CFU per gram of tissue were then calculated. Organs were embedded, sectioned, and stained with Gomori’s methenamine silver (GMS) staining kit for histopathological examination.

### RNA-Seq.

The initial inoculum of the T. asahii CBS 2479 cells were adjusted to a 0.5 McFarland standard in 20 mL YPD medium and cultured for 12 h at 35°C with shaking at 120 rpm. The treated group was then exposed to allicin at a final concentration of 256 μg mL^−1^. The cells were collected and washed three times with sterile PBS after 8 h treatment at 30°C. The samples were then quickly frozen in liquid nitrogen and stored at −80°C until required. The untreated T. asahii cells served as the control. Three independent cultures were used for RNA-Seq analysis. The transcriptome experiment was performed by Gene Denovo Biotechnology Co. (Guangzhou, China) with the following steps: total RNA extraction, mRNA enrichment by oligo(dT), RNA fragmentation, random-hexamer-primed cDNA synthesis, size selection, PCR amplification, and sequencing on an Illumina HiSeq 2500 (60).

The paired-end clean reads were aligned to the T. asahii strain CBS 2479 reference genome (https://www.ncbi.nlm.nih.gov/genome/?term=CBS+2479) using HISAT2.2.4. An FPKM (fragments per kilobase of transcript per million mapped reads) value was calculated to normalize gene expression for comparing samples. The DESeq2 software was used to identify DEGs between allicin-exposed and control groups based on an FDR of less than 0.05 and an absolute FC of ≥1.5.

To investigate the biological functions of DEGs, GO (http://geneontology.org/) enrichment analysis and KEGG (https://www.genome.jp/kegg/) pathway enrichment analysis were performed. PHI-base (http://www.phi-base.org/) analysis was performed to explore the phenotypes involved in pathogenicity and virulence among the DEGs.

### qRT-PCR analysis.

To verify the results of RNA-Seq, five genes were randomly selected to confirm their expression by qRT-PCR analysis. The cDNA was synthesized by reverse transcription using the HiScript II Q RT SuperMix for qPCR (+gDNA wiper) kit (Vazyme, Nanjing, China). The sequences of primers are listed in Table S7. PCR was then performed using the ChamQ SYBR qPCR master mix (high ROX premixed) kit (Vazyme, Nanjing, China) and a TL988 real-time PCR system (Tianlong, Xian, China). The reaction was conducted under the following conditions: 95°C for 90 s, followed by a total of 40 cycles of denaturation at 95°C for 5 s, annealing at 60°C for 15 s, and extension at 72°C for 20 s. The relative quantification of gene expression was computed using the 2^−ΔΔ^*^CT^* method and 18S rRNA as an internal reference. For qRT-PCR experiments, three biological replicates were carried out.

### Statistical analysis.

Statistical analyses were carried out using the GraphPad Prism 8.3.0 (GraphPad Software, San Diego, CA, USA) and the results were expressed as means and standard deviations (SD). Survival data were assessed by the Kaplan-Meier curve with the log-rank chi square test. Statistical differences between multiple groups were compared using one-way analysis of variance (ANOVA) followed by Tukey’s posttest. Statistical significance was set at probability (*P*) values of less than 0.05, 0.01, or 0.001.

### Data availability.

The data sets presented in this study have been deposited in the National Genomics Data Center (NGDC: https://ngdc.cncb.ac.cn/) and are accessible through GSA accession number CRA009653.
