# Supplementary material for: Antifungal Activity and Potential Action Mechanism of Allicin against Trichosporon asahii
Source: Microbiol Spectr. 2023 May 18;11(3):e00907-23. doi: 10.1128/spectrum.00907-23 (PMC10269704; doi:10.1128/spectrum.00907-23)
Supplement: Supplemental file 1 — Supplemental material. Download spectrum.00907-23-s0001.pdf, PDF file, 0.3 MB [file spectrum.00907-23-s0001.pdf]

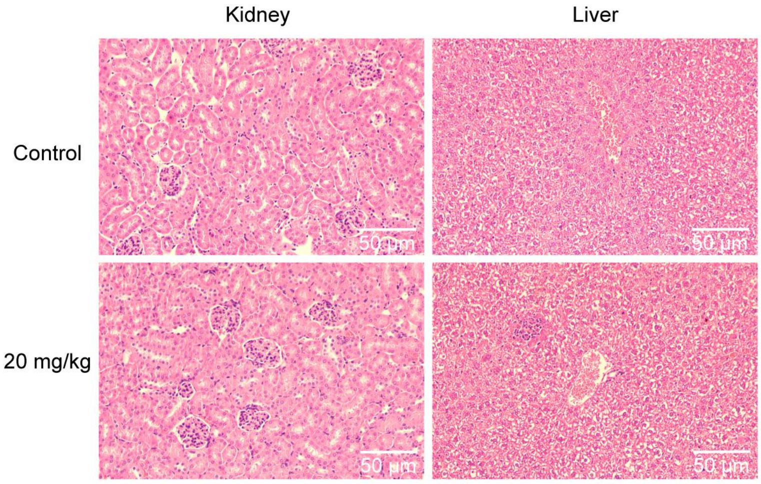

Supplementary Figure S1: Representative micrographs of HE stained kidney and liver in uninfected mice. Compared to controls, 20 $mgkg^{-1}$ allicin treatment did not lead to visceral injury in uninfected mice, indicating its safety. Magnification: 200×.
